# Microcystic, elongated, and fragmented (MELF) pattern of myometrial invasion in grade 1–2 endometrioid endometrial carcinoma: clinicopathological associations, molecular profile, and prognostic significance under FIGO 2023 staging

**DOI:** 10.3389/fonc.2026.1891878

**Published:** 2026-08-13

**Authors:** Hikmet Tunç Timur, Sefa Kurt, Rümeysa Belen Gümüş, Oğuz Arslan, Zeynep Bayramoğlu, Buket Timur, Haluk Emre Özen

**Affiliations:** 1 Department of Obstetrics and Gynecology, Dokuz Eylül University School of Medicine, İzmir, Türkiye; 2 Department of Pathology, Dokuz Eylül University School of Medicine, İzmir, Türkiye; 3 Department of Pathology, Izmir Ataturk Research and Training Hospital, İzmir, Türkiye

**Keywords:** cervical stromal invasion, endometrioid endometrial carcinoma, lymphovascular space invasion, MELF pattern, molecular classification, myometrial invasion

## Abstract

**Background:**

The microcystic, elongated, and fragmented (MELF) pattern of myometrial invasion is a morphological feature of endometrioid endometrial carcinoma (EEC) with debated prognostic significance. Most prior studies evaluated MELF across heterogeneous histologic grades and predate the FIGO 2023 staging system. We assessed MELF prevalence in a uniform Grade 1–2 EEC cohort and its clinicopathological, molecular, and survival associations.

**Methods:**

We retrospectively reviewed 112 patients with Grade 1–2 EEC surgically treated at a tertiary center (2015–2023). MELF status was determined by pathology re-review, and molecular profiling used immunohistochemistry (IHC) for mismatch repair (MMR) proteins, p53, and hormone receptors. Associations were assessed with Fisher’s exact and Mann–Whitney U tests; survival was analyzed by Kaplan–Meier, log-rank, and Cox regression with Firth penalization.

**Results:**

MELF was identified in 43/112 patients (38.4%). MELF-positive tumors were significantly associated with deep myometrial invasion (65.1% vs. 24.6%; OR 5.71, 95% CI 2.44–13.35), lymphovascular space invasion (LVSI: 58.1% vs. 11.6%; OR 10.59, 95% CI 4.01–27.95), cervical stromal invasion (32.6% vs. 10.1%; OR 4.28, 95% CI 1.55–11.82; all p ≤ 0.005), and larger tumor size (median 3.7 vs. 3.0 cm; p=0.015). MELF was not associated with deficient mismatch repair (OR 1.48, p=0.39) or p53 mutant-type expression (OR 0.96, p=1.00). At a median follow-up of 54.5 months (9 deaths), MELF-positive patients showed numerically worse overall survival that did not reach statistical significance (log-rank p=0.059). On Firth-penalized Cox regression, deep myometrial invasion was the only significant univariate predictor (HR 7.12, p=0.036); the MELF survival signal attenuated to near-unity when depth of invasion was added.

**Conclusions:**

In Grade 1–2 EEC, MELF is a frequent pattern (38.4%) that reliably flags aggressive local invasion, including cervical stromal invasion relevant to FIGO 2023 upstaging. Its survival association was largely shared with depth of myometrial invasion rather than independent of it; MELF is therefore best regarded as a morphological marker of an aggressive phenotype rather than an independent prognostic factor. Being independent of mismatch repair and p53 status, MELF may aid risk assessment within the presumed no-specific-molecular-profile (NSMP-like) subgroup. Routine pathological reporting of MELF can prompt assessment of depth of invasion and high-risk features.

## 1 Introduction

Endometrial cancer is the most common gynecologic malignancy in developed countries, with endometrioid endometrial carcinoma (EEC) accounting for approximately 80% of cases (1). Grade 1 and 2 EEC typically carry a favorable prognosis; however, a subset of patients experience disease recurrence, metastasis, and cancer-related death despite apparently low-risk histology (2, 3).

The microcystic, elongated, and fragmented (MELF) pattern of myometrial invasion was first described by Murray et al. in 2003 as a distinctive morphological alteration at the invasive front of low-grade EEC (4). MELF is characterized by dilated glands with attenuated epithelium, elongated cords of tumor cells, and fragmented single cells or clusters within a fibromyxoid stromal reaction (4, 5). Immunophenotypic studies have demonstrated features consistent with epithelial–mesenchymal transition (EMT), including downregulation of E-cadherin and hormone receptors at MELF foci and upregulation of vimentin (6).

The prognostic significance of MELF remains debated. Several studies have reported associations with deep myometrial invasion, lymphovascular space invasion (LVSI), and lymph node metastasis (LNM) (7–11). A recent meta-analysis by Zhou et al. (2025), pooling 5,587 patients from 18 studies, found a significant association with LNM (OR 3.52) and worse overall survival on univariate analysis (HR 2.31), although the survival association was not independent in multivariable analysis (12). Conversely, van den Heerik et al. (2022) reported no independent impact on recurrence in the PORTEC-1/-2 trials and recommended against routine assessment (13). A more recent systematic review by Jia et al. (2025), pooling 16 studies, confirmed significant associations of MELF with deep myometrial invasion, LVSI, LNM, cervical stromal involvement, and elevated FIGO stage (14).

A critical limitation of existing literature is the heterogeneous study populations, often including multiple histologic subtypes and grades, which dilutes the denominator and potentially underestimates MELF prevalence. Furthermore, MELF has not been adequately studied within the framework of the updated FIGO 2023 staging system, which now incorporates molecular classification (POLEmut, dMMR, NSMP, p53abn) as a key prognostic parameter (15, 16).

In this study, we aimed to: (1) determine the prevalence of MELF in a homogeneous cohort of Grade 1–2 EEC; (2) evaluate associations with clinicopathological features including cervical stromal invasion under FIGO 2023 staging; (3) assess the relationship between MELF and IHC-based molecular markers (MMR proteins, p53); and (4) evaluate the prognostic significance of MELF for overall survival.

## 2 Materials and methods

### 2.1 Study population

This retrospective study included 112 patients diagnosed with Grade 1 or Grade 2 EEC who underwent primary surgical treatment at Dokuz Eylül University Hospital, İzmir, Türkiye, between January 2015 and December 2023. Patients were identified through the institutional pathology database. Inclusion criteria were: (i) histologically confirmed endometrioid endometrial carcinoma, (ii) FIGO Grade 1 or 2, and (iii) availability of hysterectomy specimen for pathological re-review. Patients who had received neoadjuvant chemotherapy or radiation therapy were excluded. The study was approved by the institutional ethics committee. (Dokuz Eylul University Ethics Committee for Non-Invasive Studies Decision No:2025/26-12).

### 2.2 Pathological assessment

All available hematoxylin and eosin–stained slides from each hysterectomy specimen were systematically re-reviewed by an experienced gynecologic pathologist, with identification of the MELF pattern as a pre-specified primary endpoint rather than an incidental observation. MELF was defined according to the established morphological criteria of Murray et al. (4): microcystic, elongated, or fragmented glands lined by flattened or attenuated epithelium, and/or detached single tumor cells or small cell clusters at the deep invasive front, characteristically accompanied by a fibromyxoid and/or inflamed stromal reaction. A case was classified as MELF-positive when at least one unequivocal MELF focus was identified, consistent with the presence-based definition used in prior studies; cases lacking these features were classified as MELF-negative. MELF status was analyzed as a binary variable (positive vs. negative). Cases considered diagnostically equivocal for MELF by the primary reviewer (n=45) were independently re-evaluated by a second gynecologic pathologist; the second reviewer concurred with the initial classification in 43 cases and reclassified 2 equivocal cases as MELF-positive. Final MELF status was assigned by consensus. Additional pathological features recorded included: histologic grade (FIGO), depth of myometrial invasion (MI; <50% vs. ≥50%), LVSI status, cervical stromal invasion, adnexal involvement, lymph node status and tumor size (maximum dimension).

### 2.3 Immunohistochemistry and molecular profiling

Immunohistochemistry was performed on formalin-fixed, paraffin-embedded tissue sections using the following antibodies: MLH1 (clone ES05), PMS2 (clone EP51), MSH2 (clone FE11), MSH6 (clone EP49), p53 (clone DO-7), estrogen receptor (ER; clone SP1), and progesterone receptor (PR; clone PgR636). Complete loss of nuclear staining in tumor cells with retained expression in internal controls (stromal cells, lymphocytes) was interpreted as deficient for each MMR protein. The p53 staining pattern was classified as wild-type (heterogeneous staining) or mutant-type (diffuse strong overexpression or complete absence of staining). Based on IHC results, tumors were classified as: (i) mismatch repair deficient (dMMR) if any MMR protein was lost, or (ii) mismatch repair proficient (pMMR) with either p53 mutant or p53 wild-type pattern. POLE mutation testing was not performed in this study.

### 2.4 Staging

All patients were staged according to the FIGO 2023 staging system for endometrial cancer (16). The updated staging criteria incorporate histologic type, substantial LVSI, cervical stromal invasion, and molecular classification. As POLE sequencing was not available, the molecular annotation (“m” subscript) was applied only for cases with identifiable dMMR or p53abn status.

### 2.5 Statistical analysis

Categorical associations with MELF status were assessed with Fisher’s exact test (odds ratios [OR] with 95% confidence intervals [CI]); continuous variables were compared with the Mann–Whitney U test. Overall survival (OS) was defined as the time from surgery to death from any cause or last follow-up, estimated by the Kaplan–Meier method and compared with the log-rank test. Median follow-up was estimated by the reverse Kaplan–Meier method. Cox proportional hazards regression was used for univariate and multivariable analyses; the primary multivariable model included MELF status, FIGO stage, and LVSI. Deep myometrial invasion, being correlated with MELF and a probable mediator of its effect, was excluded from this model to avoid over-adjustment and assessed separately.

Given the small number of deaths (n=9), Firth-penalized Cox regression (17) was pre-specified as a sensitivity analysis for all models, with profile-likelihood 95% CIs and likelihood-ratio test p-values; multivariable survival results are interpreted as exploratory. The proportional hazards assumption was assessed using scaled Schoenfeld residuals under four time transforms. Analyses were performed in Python 3.12 (SciPy, lifelines, firthmodels v0.7.2, pandas) and R 4.3 (survival package, for the Schoenfeld test); firthmodels reproduces the reference R coxphf implementation. A two-sided p-value <0.05 was considered statistically significant.

The independence of the MELF–cervical stromal invasion association was evaluated using multivariable logistic regression (outcome, cervical stromal invasion; covariates, MELF, deep myometrial invasion, tumor size, and LVSI), with Firth-penalized logistic regression as a sensitivity analysis. Analyses were complete-case; tumor-size data were missing for 8 of 112 patients (7.1%), with missingness unrelated to MELF status (p=0.15). Nodal assessment, when performed, was by systematic lymphadenectomy; sentinel-node biopsy and cytokeratin ultrastaging were not used. Adjuvant treatment (external beam radiotherapy, vaginal brachytherapy, and chemotherapy) was abstracted from medical records and categorized into mutually exclusive groups (chemotherapy with or without radiotherapy; external beam radiotherapy with or without brachytherapy; brachytherapy alone; or none), compared using Fisher’s exact test.

## 3 Results

### 3.1 Patient characteristics

The study cohort comprised 112 patients with Grade 1–2 EEC. The median age at diagnosis was 62 years (range 39–84; IQR 54–69). Most patients were postmenopausal (86.6%, 97/112). The most common presenting symptom was postmenopausal bleeding (75.9%). Hypertension was the most common comorbidity (58.9%), followed by diabetes mellitus (29.5%). Grade 1 tumors accounted for 62.5% (n=70) and Grade 2 for 37.5% (n=42). Surgical staging including lymph node assessment was performed in 83.0% of patients, while 17.0% underwent hysterectomy with bilateral salpingo-oophorectomy only.

Regarding FIGO 2023 staging, 72.3% were Stage I (Stage IA2: 49.1%, Stage IB: 23.2%), 17.9% Stage II, and 9.8% Stage III–IV. The median follow-up was 54.5 months (IQR 30.5–90.5; range 6.5–126.5). During the follow-up period, 9 patients (8.0%) died. Baseline clinicopathological characteristics stratified by MELF status are summarized in **Table 1**.

**Table 1:** Baseline clinicopathological characteristics of the study cohort stratified by MELF status.

Characteristic	All (n=112)	MELF+ (n=43)	MELF− (n=69)	P-value
Age, years				
Median (IQR)	62 (54–69)	62 (56–68)	62 (54–69)	0.66^+^
Range	39–84	—	—	
Age group, n (%)				
<50	11 (9.8)	2 (4.7)	9 (13.0)	
50–59	40 (35.7)	16 (37.2)	24 (34.8)	
60–69	40 (35.7)	16 (37.2)	24 (34.8)	
≥70	21 (18.8)	9 (20.9)	12 (17.4)	
**Menopausal status, n (%)**				0.78
Postmenopausal	97 (86.6)	38 (88.4)	59 (85.5)	
Premenopausal	15 (13.4)	5 (11.6)	10 (14.5)	
Comorbidities, n (%)				
Hypertension	66 (58.9)	27 (62.8)	39 (56.5)	0.56
Diabetes mellitus	33 (29.5)	16 (37.2)	17 (24.6)	0.20
None	20 (17.9)	6 (14.0)	14 (20.3)	0.46
Presenting symptom, n (%)				
Postmenopausal bleeding	85 (75.9)	—	—	
Abnormal uterine bleeding	14 (12.5)	—	—	
Other	13 (11.6)	—	—	
Histologic grade, n (%)				0.16
G1	70 (62.5)	23 (53.5)	47 (68.1)	
G2	42 (37.5)	20 (46.5)	22 (31.9)	
FIGO 2023 stage, n (%)				
I	81 (72.3)	25 (58.1)	56 (81.2)	
II	20 (17.9)	12 (27.9)	8 (11.6)	
III	10 (8.9)	6 (14.0)	4 (5.8)	
IV	1 (0.9)	0 (0.0)	1 (1.4)	
**Tumor size (cm)**				**0.015^+^**
Median (IQR)	3.3 (2.0–4.5)	3.7 (3.0–5.0)	3.0 (2.0–4.0)	
Surgical procedure, n (%)				
Surgical staging¶	93 (83.0)	—	—	
Hysterectomy + BSO only	19 (17.0)	—	—	
Follow-up, months				
Median (IQR)	54.5 (30.5–90.5)	—	—	0.97^†^
Death, n (%)	9 (8.0)	6 (14.0)	3 (4.3)	0.08

IQR, interquartile range; BSO, bilateral salpingo-oophorectomy; “—” denotes subgroups not separately tabulated. P-values from Fisher’s exact test unless marked. ^+^ Mann–Whitney U test. ¶ Surgical staging refers to hysterectomy with bilateral salpingo-oophorectomy plus pelvic and/or para-aortic lymph node assessment (sentinel or systematic). ^†^reverse-Kaplan-Meier log-rank.

Bold values indicate statistical significance (p < 0.05).

### 3.2 MELF prevalence and clinicopathological associations

MELF pattern was identified in 43 of 112 patients (38.4%). The associations between MELF status and clinicopathological features are presented in **Table 2**. MELF-positive tumors were significantly more likely to exhibit deep myometrial invasion (>50%): 65.1% (28/43) vs. 24.6% (17/69) in MELF-negative tumors (OR 5.71, 95% CI 2.44–13.35, p<0.001). The strongest association was with LVSI: 58.1% (25/43) vs. 11.6% (8/69) (OR 10.59, 95% CI 4.01–27.95, p<0.001).

**Table 2:** Association between MELF pattern and clinicopathological features.

Feature	MELF+ (n=43)	MELF− (n=69)	OR (95% CI)	p-value
Deep MI (>50%)	28 (65.1%)	17 (24.6%)	5.71 (2.44–13.35)	**<0.001**
LVSI	25 (58.1%)	8 (11.6%)	10.59 (4.01–27.95)	**<0.001**
Cervical stromal invasion	14 (32.6%)	7 (10.1%)	4.28 (1.55–11.82)	**0.005**
LN metastasis	9 (20.9%)	8 (11.6%)	2.02 (0.71–5.71)	0.19
FIGO Stage III–IV	6 (14.0%)	5 (7.2%)	2.08 (0.59–7.30)	0.33
Mortality	6 (14.0%)	3 (4.3%)	3.57 (0.84–15.11)	0.08

Fisher’s exact test. Bold p-values indicate statistical significance (p<0.05). MI, myometrial invasion; LVSI, lymphovascular space invasion; LN, lymph node; OR, odds ratio; CI, confidence interval.

Bold values indicate statistical significance (p < 0.05).

Cervical stromal invasion was significantly more common in MELF-positive patients: 32.6% (14/43) vs. 10.1% (7/69) (OR 4.28, 95% CI 1.55–11.82, p=0.005). MELF-positive tumors were also significantly larger than MELF-negative tumors (median 3.7 cm [IQR 3.0–5.0] vs. 3.0 cm [IQR 2.0–4.0]; Mann–Whitney p=0.015).

To determine whether the association between MELF and cervical stromal invasion was independent of the broader invasive phenotype, we performed a multivariable logistic regression with cervical stromal invasion as the outcome and MELF, deep myometrial invasion, tumor size, and LVSI as covariates (n=104 with complete tumor-size data; 21 events; **Table 3**). After adjustment, MELF was no longer independently associated with cervical stromal invasion (adjusted OR 1.97, 95% CI 0.47–8.26, p=0.36); the independent predictors were tumor size (adjusted OR 2.26 per cm, 95% CI 1.48–3.47, p<0.001) and deep myometrial invasion (adjusted OR 9.10, 95% CI 1.65–50.10, p=0.011), whereas LVSI was not independent (adjusted OR 0.68, p=0.62). Results were materially unchanged using Firth-penalized logistic regression (MELF OR 1.86, 95% CI 0.47–7.43, p=0.22). Thus, the univariate MELF–cervical stromal invasion association reflects a shared, more invasive phenotype rather than an effect independent of tumor size and depth of invasion.

**Table 3:** Multivariable logistic regression for cervical stromal invasion (n=104; 21 events).

Predictor	Adjusted OR (95% CI)	p	Firth OR (95% CI)	p
MELF (present vs absent)	1.97 (0.47–8.26)	0.36	1.86 (0.47–7.43)	0.22
Deep myometrial invasion (>50%)	9.10 (1.65–50.10)	0.011	7.29 (1.61–40.80)	0.007
Tumor size (per 1 cm)	2.26 (1.48–3.47)	<0.001	2.06 (1.46–3.25)	<0.001
LVSI (present vs absent)	0.68 (0.14–3.23)	0.62	0.72 (0.15–3.07)	0.39

Multivariable (standard maximum-likelihood) and Firth-penalized logistic regression; outcome, cervical stromal invasion. OR, odds ratio; CI, confidence interval; LVSI, lymphovascular space invasion.

**Figure 1:**
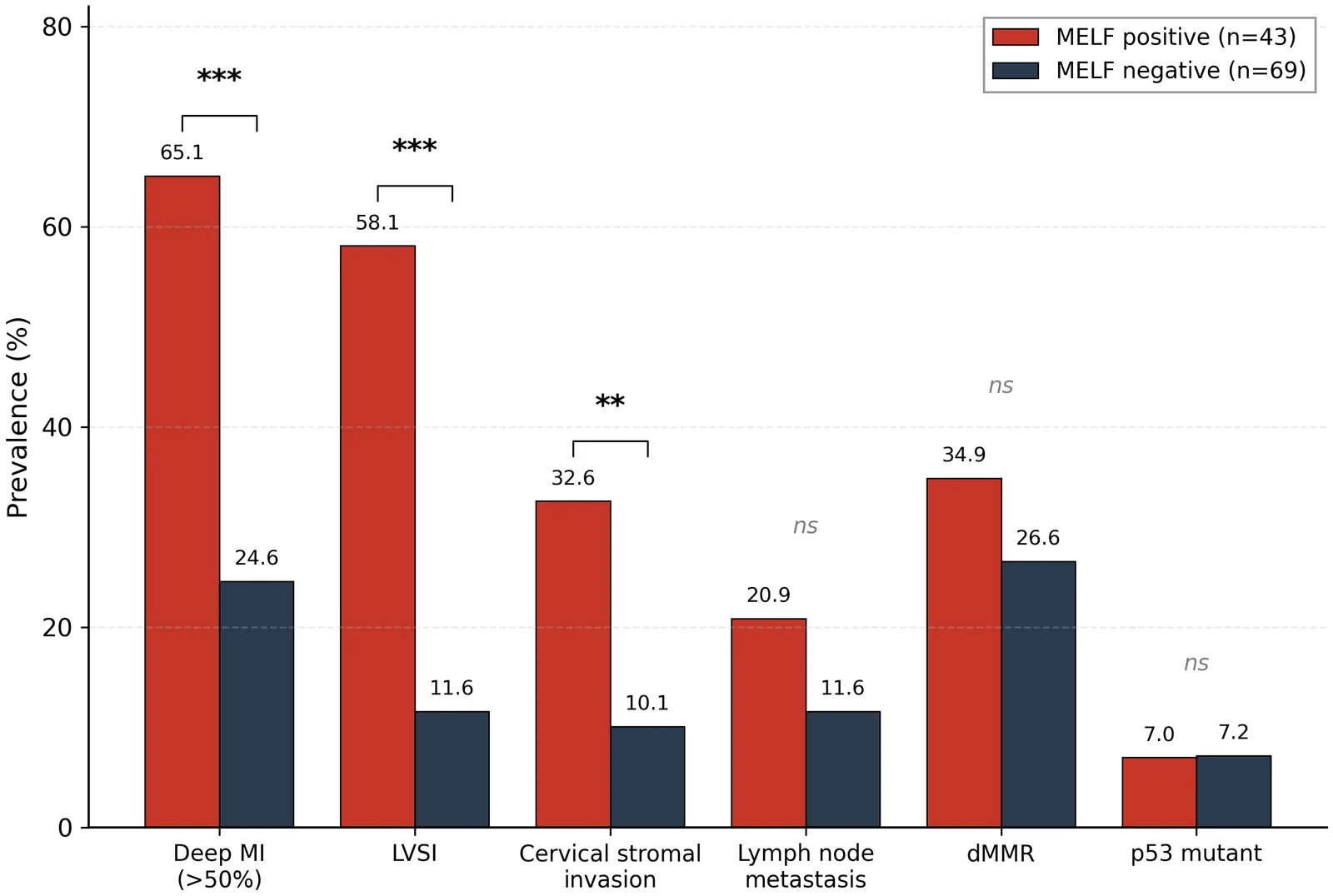
Bar chart comparing the prevalence of key clinicopathological features between MELF-positive (red, n=43) and MELF-negative (navy, n=69) tumors. Numerical values above each bar represent percentage prevalence. Statistical significance from Fisher’s exact test: ***p<0.001, **p<0.01; ns, not significant. The dMMR percentage is based on cases with interpretable mismatch repair immunohistochemistry (n=107). MI, myometrial invasion; LVSI, lymphovascular space invasion; dMMR, deficient mismatch repair.

Lymph node metastasis was numerically more frequent in MELF-positive patients (20.9% [9/43] vs. 11.6% [8/69]; OR 2.02, 95% CI 0.71–5.71, p=0.19), although this difference did not reach statistical significance and the confidence interval was wide. Advanced-stage disease (FIGO III–IV) was numerically more common in MELF-positive patients (14.0% vs. 7.2%; OR 2.08, p=0.33) but did not reach statistical significance. Mortality was numerically higher in MELF-positive patients (14.0% [6/43] vs. 4.3% [3/69]; OR 3.57, 95% CI 0.84–15.11, p=0.08), but the difference was not statistically significant. A graphical comparison of key clinicopathological features by MELF status is shown in **Figure 1**.

Nodal assessment (systematic lymphadenectomy) was performed in 83.0% (93/112) of patients and did not differ between MELF-positive (81.4%, 35/43) and MELF-negative (84.1%, 58/69) patients (p=0.80). Restricting the nodal-metastasis comparison to surgically staged patients was consistent with the full cohort (22.9% [8/35] vs. 12.1% [7/58]; OR 2.16, p=0.24), arguing against substantial verification bias; sentinel-node biopsy and cytokeratin ultrastaging were not used.

Adjuvant therapy differed by MELF status (**Table 4**): MELF-positive patients were less often managed by observation alone (30.2% vs. 49.3%) and more often received chemotherapy (34.9% vs. 15.9%; p=0.037), with the overall distribution differing between groups (p=0.037).

**Table 4:** Adjuvant therapy by MELF status.

Adjuvant therapy	All (n=112)	MELF+ (n=43)	MELF− (n=69)
None/observation	47 (42.0%)	13 (30.2%)	34 (49.3%)
Vaginal brachytherapy only	0	0	0
External beam RT ± VBT	39 (34.8%)	15 (34.9%)	24 (34.8%)
Chemotherapy (± RT)	26 (23.2%)	15 (34.9%)	11 (15.9%)

Categories are mutually exclusive; overall distribution compared by Fisher–Freeman–Halton test (p=0.037); chemotherapy 34.9% vs. 15.9% (p=0.037); any adjuvant therapy 69.8% vs. 50.7% (p=0.052). RT, radiotherapy; VBT, vaginal brachytherapy.

### 3.3 Molecular profile

IHC-based molecular classification was available for 107 patients (95.5%). Overall, 70.1% (75/107) were pMMR and 29.9% (32/107) were dMMR. Among dMMR cases, the most common pattern was MLH1 ± PMS2 loss, followed by isolated PMS2 loss, MSH2/MSH6 loss, and isolated MSH6 loss.

MELF status was not significantly associated with dMMR: 34.9% (15/43) of MELF-positive vs. 26.6% (17/64) of MELF-negative patients with available MMR data (OR 1.48, 95% CI 0.63–3.46, p=0.39). Similarly, p53 mutant-type expression was observed in 7.0% (3/43) of MELF-positive and 7.2% (5/69) of MELF-negative patients (OR 0.96, 95% CI 0.22–4.24, p=1.00). Among patients with interpretable IHC, those without mismatch repair deficiency or aberrant p53 expression were considered to have an NSMP-like profile; definitive NSMP classification was not possible because POLE sequencing was not performed. These findings indicate that MELF is a morphological phenotype independent of MMR and p53 status; because POLE sequencing was not performed, independence from a fully defined molecular classification cannot be asserted.

Hormone receptor expression was nearly universal: ER positivity was 100% and PR positivity was 99.1% (111/112), consistent with the low-grade endometrioid histology of the cohort. The molecular findings are detailed in **Table 5**.

**Table 5:** Molecular profile (immunohistochemistry) by MELF status.

Marker	MELF+	MELF−	OR (95% CI)	p-value
dMMR (any loss)¹	15/43 (34.9%)	17/64 (26.6%)	1.48 (0.63–3.46)	0.39
p53 mutant-type	3/43 (7.0%)	5/69 (7.2%)	0.96 (0.22–4.24)	1.00
ER positive	43/43 (100%)	69/69 (100%)	—	—
PR positive	43/43 (100%)	68/69 (98.6%)	—	1.00

¹ Denominator for dMMR analysis reflects cases with interpretable MMR immunohistochemistry. ER, estrogen receptor; PR, progesterone receptor.

### 3.4 Survival analysis

**Figure 2:**
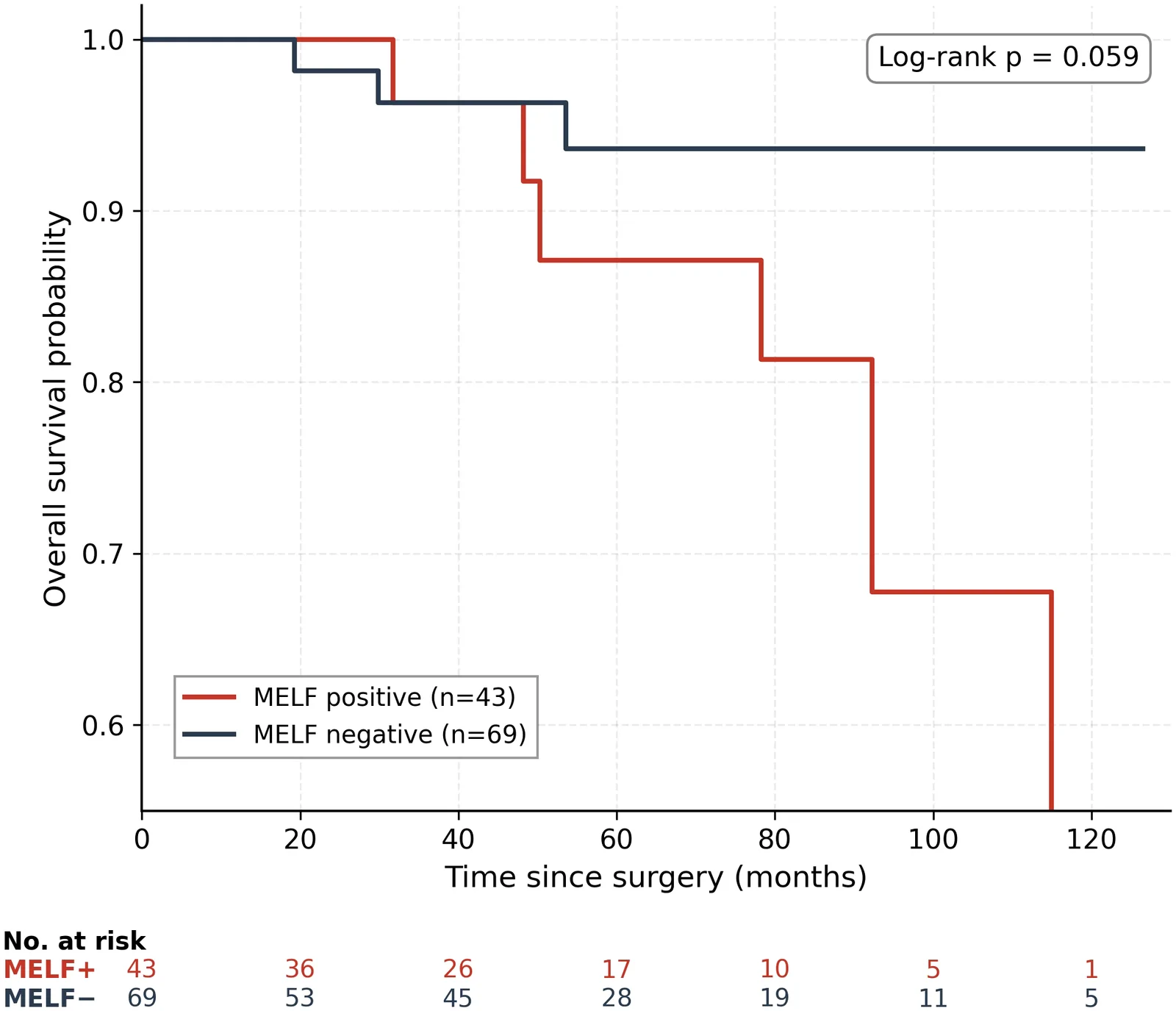
Kaplan–Meier overall survival curves stratified by MELF status in 112 patients with Grade 1–2 endometrioid endometrial carcinoma. MELF-positive patients (red, n=43) showed numerically worse survival than MELF-negative patients (navy, n=69), a difference that was not statistically significant. Log-rank p=0.059.

At a median follow-up of 54.5 months, 9 patients had died (8.0%). MELF-positive patients showed numerically worse overall survival (log-rank p=0.059; **Figure 2**), a difference that did not reach statistical significance. Given only nine deaths (events-per-variable ratio of 3), these survival analyses are exploratory. On univariate Cox regression, MELF-positive status was not significantly associated with mortality (HR 3.51, 95% CI 0.87–14.07); deep myometrial invasion was the only significant predictor (HR 10.05, 95% CI 1.26–80.40), while LVSI and stage III–IV showed non-significant trends (**Table 6**).

**Table 6:** Cox proportional hazards regression for overall survival.

Variable	Univariate HR	95% CI	p	Adjusted HR¹	95% CI	p
MELF positive	3.51	0.87–14.07	0.077	2.28	0.42–12.34	0.34
Deep myometrial invasion (>50%)	10.05	1.26–80.40	0.030	—	—	—
LVSI positive	3.36	0.84–13.51	0.088	2.03	0.37–11.13	0.42
Stage III–IV	2.00	0.24–16.69	0.52	1.47	0.17–12.57	0.73

¹ Multivariable Cox model adjusted for MELF status, LVSI (inclusive coding), and FIGO 2023 Stage III–IV. Deep myometrial invasion was excluded from this primary model because it is correlated with MELF (φ=0.40) and may lie on the causal pathway between the MELF invasive phenotype and death, such that its inclusion would constitute over-adjustment. An exploratory model additionally including deep myometrial invasion (Results, section 3.4) shows the MELF hazard ratio attenuating to near-unity (Firth-penalized aHR 1.18, 95% CI 0.24–7.07) while deep myometrial invasion retains a strong association (Firth-penalized aHR 7.24, 95% CI 0.98–80.78). The estimates shown use standard partial-likelihood Cox regression with Wald-based 95% CIs. Pre-specified sensitivity analyses using Firth-penalized Cox regression (17), which provides bias-corrected estimates and profile-likelihood CIs more appropriate for sparse-events settings, are reported in Methods (section 2.5) and Results (section 3.4). Under Firth penalization, univariate HRs were: MELF 3.26 (PL 95% CI 0.92–13.72, p=0.092), deep MI 7.12 (PL 95% CI 1.60–66.90, p=0.036, the only significant predictor), LVSI 3.12 (PL 95% CI 0.88–13.17, p=0.105), Stage III–IV 2.76 (PL 95% CI 0.29–13.23, p=0.30); the Firth-penalized multivariable aHRs (with profile-likelihood 95% CI and LRT p-values) were MELF 2.12 (0.44–12.14, p=0.37), LVSI 1.93 (0.38–10.98, p=0.44), Stage III–IV 1.98 (0.20–9.80, p=0.49). The proportional hazards assumption showed no significant violation for LVSI or Stage III–IV under any time transform; results for MELF were borderline and transform-dependent (scaled Schoenfeld p=0.040–0.107, significant only under the rank transform). HR, hazard ratio; CI, confidence interval; LVSI, lymphovascular space invasion; PL, profile-likelihood; LRT, likelihood ratio test.

In the primary multivariable model (MELF, LVSI, and stage), the MELF adjusted HR was 2.28 (95% CI 0.42–12.34) and remained non-significant. Deep myometrial invasion was excluded from this model to avoid over-adjustment (see Methods); in an exploratory Firth-penalized model that added it, the MELF hazard ratio attenuated to near-unity (aHR 1.18) while deep invasion retained a strong association (aHR 7.24), indicating that the survival signal attributed to MELF was largely shared with depth of invasion rather than independent of it. Firth-penalized sensitivity analyses were concordant throughout (**Table 6**).

In robustness checks, the proportional hazards assumption was satisfied for LVSI and stage III–IV but borderline for MELF, reaching significance only under the rank transform (p=0.040). Follow-up time was balanced between groups (p=0.97), and tumor-size data missing in 8 of 112 patients were unrelated to MELF status (p=0.15), with a complete-case analysis directionally consistent.

Five recurrences were observed (4.5%): 7.0% (3/43) in MELF-positive versus 2.9% (2/69) in MELF-negative patients (p=0.37). Given only five events, recurrence-free survival was not formally modeled (exploratory log-rank p=0.31), and disease-specific survival could not be assessed because cause of death was not recorded.

## 4 Discussion

This study demonstrates that the MELF pattern of myometrial invasion is a frequent finding in Grade 1–2 EEC (38.4%) and serves as a robust morphological marker of aggressive local invasion behavior, with strong associations with deep myometrial invasion, LVSI, and cervical stromal invasion. Importantly, MELF was independent of IHC-based molecular classification, indicating that it captures a morphological dimension of tumor behavior not reflected in the molecular subgroups and may therefore be useful for risk assessment within the NSMP subgroup, where molecular markers do not further stratify risk.

Our MELF prevalence of 38.4% is substantially higher than that reported in major published series, including the pooled estimate of 20.4% in the Zhou et al. meta-analysis (12), 13.1% observed in the PORTEC-1/-2 trials (13), 23% in the Grade 1–2 subset of Altindağ et al. (n=217) (11), and 12.9% in the pure FIGO Grade 1 EEC cohort of Joehlin-Price et al. (n=464) (7). Notably, this discrepancy is not explained by population heterogeneity, since Joehlin-Price restricted enrolment to Grade 1 EEC only and still reported a substantially lower prevalence. The most likely explanations are therefore methodological. First, in routine diagnostic sign-out, MELF is not a mandated reporting element and may not be routinely sought; in the present study, by contrast, every specimen underwent dedicated whole-slide re-review with MELF identification as a pre-specified endpoint, a design that increases detection of focal MELF that would otherwise go unrecorded. Second, MELF assessment is known to be subject to interobserver variability, and a single pathologist’s threshold for calling MELF may differ from multi-reader consensus. Third, MELF prevalence varies considerably across the literature even among methodologically similar studies, reflecting the absence of standardized, quantitative diagnostic criteria. We acknowledge that overestimation of MELF prevalence is a possible limitation of our methodology, and that interobserver agreement and standardized criteria would strengthen comparability across studies.

The association between MELF and deep myometrial invasion (OR 5.71) and LVSI (OR 10.59) in our cohort is consistent with the literature and supports the biological concept that MELF represents a form of EMT at the tumor–stromal interface (5, 6). The particularly strong association with LVSI aligns with immunophenotypic studies demonstrating that MELF areas show enhanced migratory capacity, with PD-L1 upregulation at the invasive front facilitating immune evasion and lymphovascular entry (18).

We observed a significant univariate association between MELF and cervical stromal invasion (OR 4.28, p=0.005); on multivariable analysis, however, this was not independent of tumor size and depth of invasion (adjusted OR 1.97, p=0.36; **Table 3**), indicating that MELF flags rather than independently drives cervical stromal extension. Our contribution is to situate this association explicitly within the FIGO 2023 framework, under which cervical stromal invasion upstages disease to Stage II. Cervical involvement has been reported among MELF-associated features in the systematic review by Jia et al. (14), and our analysis extends these findings by testing its independence and situating it within contemporary staging. This association is biologically plausible: the same EMT-driven invasive phenotype that facilitates deep myometrial penetration could extend into the cervical stroma. We also observed that MELF-positive tumors were significantly larger than their MELF-negative counterparts (median 3.7 vs. 3.0 cm; p=0.015), consistent with the findings of Song et al. (19).

The lymph node metastasis rate in MELF-positive patients (20.9%) was approximately double that of MELF-negative patients (11.6%), though this difference did not reach statistical significance (OR 2.02, p=0.19). The Zhou et al. meta-analysis, with substantially greater statistical power (n=5,587), reported a highly significant OR of 3.52 for LNM (12). Our point estimate is in the same direction, and the lack of significance likely reflects limited sample size. The clinical relevance of MELF-associated nodal deposits is underscored by studies showing these are often low-volume metastases—isolated tumor cells or micrometastases that may be missed without cytokeratin immunohistochemistry ultrastaging (20).

Our finding that MELF is not associated with dMMR (OR 1.48, p=0.39) or p53 mutation (OR 0.96, p=1.00) is consistent with the PORTEC data, where 85.6% of MELF-positive cases were NSMP (13). This independence from MMR and p53 status has important implications. In the era of molecular classification, where treatment decisions are increasingly guided by the four-tier framework (POLEmut, dMMR, NSMP, p53abn) (21), MELF could serve as an additional risk stratifier within the heterogeneous NSMP group, which comprises approximately 50% of all endometrial cancers and currently lacks reliable morphological prognostic markers beyond hormone receptor status (22).

On survival analysis, MELF-positive patients showed numerically worse overall survival that did not reach statistical significance (log-rank p=0.059; univariate HR 3.51, 95% CI 0.87–14.07, p=0.077). When adjusted for stage and LVSI, the hazard ratio remained elevated but non-significant (aHR 2.28, 95% CI 0.42–12.34). However, when deep myometrial invasion was added to the model, the MELF effect attenuated to near-unity while deep invasion retained a strong association, indicating that the MELF survival signal is largely shared with depth of invasion rather than independent of it. This finding parallels the Zhou et al. meta-analysis, which reported a univariate HR of 2.31 for overall survival that attenuated and did not remain significant in multivariable analysis (12). The consistent pattern across studies—a univariate signal that attenuates after adjustment for established prognostic factors, particularly deep myometrial invasion—indicates that MELF is best understood not as an independent prognostic factor but as a readily identifiable morphological marker of an aggressive, deeply invasive phenotype. Its clinical value lies in flagging tumors that warrant careful assessment of depth of invasion, LVSI, and cervical involvement, all of which directly affect FIGO 2023 staging and adjuvant treatment decisions.

### 4.1 Clinical implications

Our findings support several clinical recommendations. First, MELF is a readily identifiable feature that may be noted in Grade 1–2 EEC hysterectomy specimens, as it flags a subset of ostensibly low-risk tumors with aggressive local invasion; however, given the retrospective single-center design, the absence of recurrence endpoints, the lack of independent prognostic significance, and the absence of external validation, prospective multicenter validation is required before MELF can inform adjuvant treatment or surgical-staging decisions. Second, the strong association with LVSI and deep invasion supports comprehensive surgical staging, including sentinel lymph node mapping with ultrastaging, even when preoperative assessment suggests low-risk disease. Third, in MELF-positive/NSMP-like patients, the combination of deep invasion, LVSI, and cervical involvement may justify adjuvant treatment (external beam radiation or vaginal brachytherapy) consistent with intermediate or high-intermediate risk classification per ESGO/ESTRO/ESP guidelines (15). Jia and Zhang recently reported that among 43 intermediate- and high-intermediate-risk MELF-positive EEC patients, those receiving chemotherapy with or without radiotherapy had significantly better disease-free survival than those who did not receive chemotherapy (p=0.047), and concluded that MELF status should be incorporated into adjuvant therapy decision-making (23).

### 4.2 Comparison with key studies

Several features distinguish our study from prior reports. First, we focused exclusively on Grade 1–2 EEC, providing a homogeneous cohort that reflects the true biological context of MELF. Second, we applied the FIGO 2023 staging system, which integrates molecular classification and recognizes substantial LVSI as a staging criterion. Third, we evaluated the MELF–cervical stromal invasion association explicitly within the FIGO 2023 staging framework and formally tested its independence in multivariable analysis. Fourth, our IHC panel including MMR proteins and p53 places MELF within the molecular classification framework, confirming its enrichment in the presumed NSMP subgroup.

When compared with the PORTEC-1/-2 analysis (13), which concluded that MELF has no clinical implications and recommended against routine assessment, our study reaches a different conclusion. The key difference is in perspective: the PORTEC endpoint was recurrence in (high-)intermediate-risk patients of all histologies, while we focused on associations with invasion features and survival in a purely Grade 1–2 EEC population. Furthermore, the PORTEC observation that MELF-positive patients had paradoxically lower 5-year recurrence (4.9% vs. 12.7%) most likely reflects strong enrichment of MELF in the NSMP molecular subgroup (85.6% of MELF-positive cases in their cohort).

### 4.3 Limitations

This study has several limitations. It is a retrospective, single-center study, which introduces selection and information bias and limits generalizability. The survival analysis is constrained by only 9 deaths (events-per-variable ratio of 3): the hazard ratios are exploratory, and although Firth-penalized regression confirmed their direction, precision is inherently limited; the proportional hazards assumption was also borderline and transform-dependent for MELF, so the hazard ratios are best read as an average effect across follow-up. Multiple associations were tested without correction for multiple comparisons, raising the possibility of type I error among borderline results — though the principal associations with deep myometrial invasion and lymphovascular space invasion (both p<0.001) survive any conventional correction. Other limitations include the absence of POLE testing (precluding full molecular classification), the binary scoring of MELF without focal/extensive grading, non-standardized adjuvant treatment, the lack of formal interobserver reproducibility assessment, and missing tumor-size data in 8 patients. Importantly, the descriptive associations in **Table 2** do not depend on survival events or the proportional hazards assumption and constitute the most robust contribution of this study.

A high proportion of cases (45/112, 40.2%) were initially considered equivocal for MELF by the primary reviewer and underwent independent second-reviewer adjudication, of which consensus reclassified only two as MELF-positive; this reflects the inherently subjective, presence-based nature of MELF assessment in the absence of standardized quantitative criteria. A formal interobserver reproducibility statistic (κ) was not calculated, and because MELF was scored as a binary variable without focus-level quantification, a threshold-based sensitivity analysis was not feasible. To allow readers to judge our diagnostic threshold, representative photomicrographs of MELF-positive foci are provided as **Supplementary Figure 1**. Nodal assessment was performed by systematic lymphadenectomy without cytokeratin ultrastaging, so low-volume metastases—disproportionately associated with MELF—may have been under-detected, which would attenuate rather than inflate the observed nodal association. Adjuvant treatment was non-standardized and more frequent in MELF-positive patients (including more chemotherapy), which may confound the survival comparison. Recurrence events were few (n=5) and cause-of-death data were unavailable, precluding adequately powered recurrence-free or disease-specific survival analyses.

### 4.4 Future directions

A multicenter validation study with a larger cohort (≥500 patients), complete four-tier molecular classification including POLE sequencing, and standardized adjuvant protocols is warranted. Additional areas of investigation include: quantitative MELF grading, CTNNB1 mutational analysis within the NSMP subgroup, PD-L1 expression at the invasive MELF front, and correlation with preoperative imaging to explore the feasibility of non-invasive MELF prediction. Integration of MELF into prospective adjuvant therapy trials should also be considered.

## 5 Conclusions

The MELF pattern is a frequent morphological finding in Grade 1–2 EEC (38.4% in this cohort) that strongly associates with deep myometrial invasion, lymphovascular space invasion, cervical stromal invasion, and larger tumor size. MELF was independent of mismatch repair and p53 status, positioning it as a complementary morphological marker within the molecular classification framework, with potential relevance for refining risk assessment in the no-specific-molecular-profile (NSMP) subgroup. On survival analysis, MELF-positive patients showed numerically worse overall survival that did not reach statistical significance, attenuated after adjustment, and was largely shared with depth of myometrial invasion; MELF is therefore best regarded as a readily identifiable marker of an aggressive, deeply invasive phenotype rather than an independent prognostic factor. Routine pathological reporting of MELF in Grade 1–2 EEC is nonetheless warranted, as it provides an easily recognized flag prompting thorough assessment of depth of invasion and other high-risk features relevant to FIGO 2023 staging. Multicenter validation incorporating POLE sequencing is the essential next step.

## Data Availability

The raw data supporting the conclusions of this article will be made available by the authors, without undue reservation.

## Supplementary material

Supplementary Figure 1 Representative photomicrographs of the microcystic, elongated, and fragmented (MELF) pattern of myometrial invasion in endometrioid endometrial carcinoma (hematoxylin and eosin). (A) Low magnification overview of the invasive front, showing irregular, angulated glands dispersed within an abundant fibromyxoid, inflamed stroma. (B) Scattered small glands and detached epithelial fragments are seen within the fibromyxoid stroma. (C, D) Higher magnification views of two separate foci showing microcystic, dilated glands lined by flattened (attenuated) epithelium with focal fragmentation, containing detached and degenerate epithelial cells within the lumina, set in a myxoid, fibroinflammatory stroma. Scale bars are shown on each panel.
